# Large-scale viral genome analysis identifies novel clinical associations between hepatitis B virus and chronically infected patients

**DOI:** 10.1038/s41598-019-46609-7

**Published:** 2019-07-19

**Authors:** Ondrej Podlaha, Edward Gane, Maurizia Brunetto, Scott Fung, Wan-Long Chuang, Calvin Q. Pan, Zhaoshi Jiang, Yang Liu, Neeru Bhardwaj, Prasenjit Mukherjee, John Flaherty, Anuj Gaggar, Mani Subramanian, Namiki Izumi, Shalimar, Young-Suk Lim, Patrick Marcellin, Maria Buti, Henry L. Y. Chan, Kosh Agarwal

**Affiliations:** 1 Gilead Sciences Inc., 333 Lakeside Drive, Foster City, CA 94404 USA; 2 Auckland Clinical Studies, Auckland, New Zealand; 3 Internal Medicine, Department of Clinical and Experimental Medicine, University of Pisa, Pisa, Italy; 4 Liver Unit, University Hospital of Pisa Hepatology Unit, University Hospital of Pisa, Pisa, Italy; 5 Toronto General Hospital, Toronto, ON Canada; 6 Kaohsiung Medical University Hospital, Kaohsiung Medical University, Kaohsiung, Taiwan; 7 Division of Gastroenterology and Hepatology, Department of Medicine, NYU Langone Health, NYU School of Medicine, New York, NY USA; 8 Department of Gastroenterology and Hepatology, Musashino Red Cross Hospital, Tokyo, Japan; 9 All India Institute of Medical Sciences, Department of Gastroenterology, New Delhi, India; 10 Department of Gastroenterology, Asan Medical Center, University of Ulsan College of Medicine, Seoul, South Korea; 11 Service d’Hépatologie, Hôpital Beaujon, Clichy, France; 12 Liver Unit, Department of Medicine, Hospital General Universitari Vall d’Hebron and Ciberehd del Instituto Carlos III, Barcelona, Spain; 13 The Chinese University of Hong Kong, Hong Kong, China; 14 Kings College Hospital, London, UK

**Keywords:** Genome informatics, Genome-wide association studies, Genomics, Hepatitis B virus, Hepatitis B

## Abstract

Despite the high global prevalence of chronic hepatitis B (CHB) infection, datasets covering the whole hepatitis B viral genome from large patient cohorts are lacking, greatly limiting our understanding of the viral genetic factors involved in this deadly disease. We performed deep sequencing of viral samples from patients chronically infected with HBV to investigate the association between viral genome variation and patients’ clinical characteristics. We discovered novel viral variants strongly associated with viral load and HBeAg status. Patients with viral variants C1817T and A1838G had viral loads nearly three orders of magnitude lower than patients without those variants. These patients consequently experienced earlier viral suppression while on treatment. Furthermore, we identified novel variants that either independently or in combination with precore mutation G1896A were associated with the transition from HBeAg positive to the negative phase of infection. These observations are consistent with the hypothesis that mutation of the HBeAg open reading frame is an important factor driving CHB patient’s HBeAg status. This analysis provides a detailed picture of HBV genetic variation in the largest patient cohort to date and highlights the diversity of plausible molecular mechanisms through which viral variation affects clinical phenotype.

## Introduction

Chronic hepatitis B virus (HBV) infection is a significant public health burden with over 290 million chronically infected individuals worldwide^1,2^. Although effective vaccines now exist to prevent HBV infection, most cases in highly endemic areas are the result of perinatal transmission leading to chronic infection, for which there is no cure.

Current clinical practice guidelines of the European Association for the Study of the Liver (EASL) recognize five phases of HBV infection determined by a combination of clinical factors such as the viral load (serum levels of HBV DNA), the presence of viral hepatitis B e antigen (HBeAg), hepatitis B s antigen (HBsAg) levels, alanine aminotransferase (ALT) levels, and the stage of liver fibrosis^3^. These clinical factors are well known to be predictive of progression to cirrhosis, hepatocellular carcinoma, and other liver-related complications, including mortality^4–8^. Since the HBV genome is the blueprint for the viral interaction with the human host, understanding what genetic factors of the virus are correlated with these clinical parameters is essential for elucidating the molecular mechanisms of the disease progression. Previous studies investigating associations between viral genetic variants and patient clinical characteristics were largely restricted to the core promoter and precore regions of the virus or sequenced only a small sample of viral clones per patient^9^. We undertook a more comprehensive analysis of associations between HBV single nucleotide variants and patient clinical characteristics by looking at the whole viral genome across a large patient cohort.

## Results

### Study populations and sample description

We performed an ultra-deep (average ~9,000x coverage) whole HBV genome sequencing of 1467 patients (1102 in discovery and 365 in validation cohort) chronically infected with HBV at baseline. The patient population contained HBV genotypes A (N = 98), B (N = 285), C (N = 716), D (N = 356), E (N = 7), and F (N = 5) with 977 HBeAg-positive and 490 HBeAg-negative patients (Table 1).

**Table 1:** Clinical characteristics of patients included in this study.

	Clinical characteristics	HBeAg Negative	HBeAg Positive
		N = 490 (33%)	N = 977 (67%)
	Age	44.90 +/− 0.50	36.89 +/− 0.36
Gender	Male	314 (64%)	616 (63%)
	Female	176 (36%)	361 (37%)
Race	White	138 (28%)	174 (18%)
	Asian	343 (70%)	788 (81%)
	Black	5 (1%)	8 (1%)
	Pacific Islander	3 (1%)	3 (0%)
	Other	1 (0%)	4 (0%)
Genotype	A	27 (6%)	71 (7%)
	B	119 (24%)	166 (17%)
	C	190 (39%)	526 (54%)
	D	150 (31%)	206 (21%)
	E	4 (1%)	3 (0%)
	F	0 (0%)	5 (1%)
	HBV DNA [log_10_ IU/mL]	5.93 +/− 0.06	7.71 +/− 0.04
	HBsAg [log10 IU/mL]	3.40 +/− 0.03	4.10 +/− 0.02
	ALT [U/L]	101.65 +/− 5.46	120.95 +/− 3.64

### Genetic determinants of viral load

**Figure 1:**
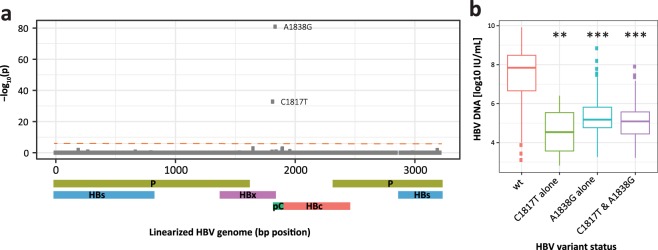
HBV variants associated with viral load. (**a**) Manhattan plot showing association (Likelihood Ratio Test *p* value, see methods) between HBV variants at a given genomic position and patient’s viral load (serum HBV DNA levels). Schematic below x axis represents HBV genome structure. P – polymerase, S – surface protein, C – core protein, pC – pre-core region, X – HBx protein. Bonferroni correction leads to a significance threshold of approximately 1.6 × 10^−6^ indicated by red dashed line. (**b**) Patients with high C1817T and A1838G variant frequency (based on 10% cutoff, see methods) had significantly lower HBV DNA levels. Wilcoxon rank sum test: *p < 0.05; **p < 0.01; ***p < 0.001.

Across the viral genome, the most significant associations were observed between the viral load and two non-synonymous variants C1817T (HBc gene Q > STOP; HBx gene C > C; likelihood ratio test [LRT] p = 1.7 × 10^−33^, Fig. 1a) and A1838G (HBc gene I > V; HBx gene: STOP > STOP; LRT p = 1.3 × 10^−81^, Fig. 1a**)**. It is well known that the transition from HBeAg positive to negative phase of HBV infection is generally marked by a reduction in viral replication and viral load^10–15^. In order to control for this confounding effect, patients’ HBeAg status was included as a covariate in our model during testing. Regardless of HBeAg status, however, C1817T and A1838G were both highly associated with viral load. The intra-patient frequency distribution of both variants across the patient cohort revealed a bimodal distribution, broadly dividing patients into low and high variant intra-patient frequency groups based on a naturally observed 10% variant frequency cutoff (Supplementary Fig. 1). At this cutoff patients with high frequency of C1817T and A1838G had median viral loads nearly three orders of magnitude lower (reduction by 2.8 log_10_IU/mL of serum HBV DNA) than patients without or with low frequency of these variants (Wilcoxon rank sum test, p < 10^−5^, Fig. 1b and Supplementary Fig. 2; see Supplemental Table 1 for mutation prevalence among HBV genotypes). To ensure that this observation was not sensitive to the naturally observed frequency cutoff, we performed identical comparisons grouping patients across a range of cutoffs (5–20%) all of which led to equivalent conclusions (Supplemental Table 2). As a result of lower viral load, patients with high frequency of C1817T and A1838G experienced earlier viral suppression while on treatment (LRT p < 1.0 × 10^−6^ for weeks 4, 8, 12, 24, and 48). Both C1817T and A1838G associations were validated in an independent patient cohort (N = 365; LRT p = 1.2 × 10^−17^ and p < 5.2 × 10^−31^, respectively; Supplementary Fig. 3). Furthermore, the suppressive effects of these variants on viral load were experimentally confirmed in a transient transfection system (Supplementary Fig. 4).

Although we identified C1817T association with viral load while controlling for HBeAg status, our findings do not exclude the possibility that this variant could have pleiotropic effects on multiple clinical factors. In fact, experimental evidence indicates that mutating the second precore codon to a stop codon results in the elimination of HBeAg expression^16^. HBeAg is the product of precore mRNA which nearly completely overlaps pregenomic (pg) RNA^17^, suggesting an effect of C1817T on both the precore mRNA and pgRNA transcription. Indeed, C1817T is additionally associated with HBeAg status in our data as well (LRT p = 2.8 × 10^−12^).

Aside from C1817T and A1838G variants, it is worth mentioning that C1653T, G1896A, and G1899A displayed the next most significant association with viral load in the discovery patient cohort (LRT p = 6 × 10^−8^, p = 6.7 × 10^−8^, and p = 2.3 × 10^−7^, respectively). Although their associations with viral load did not reach stringent *p* value cutoff (p < 1.6 × 10^−6^) in the validation cohort, their trending *p* values warrant future confirmation. Earlier studies suggest that these variants impact viral replication process via different molecular mechanisms. Specifically, C1653T alters the binding site of the CAAT enhancer-binding protein of the alpha-box within the HBV enhancer II region^18^, which is known to transcriptionally regulate the pgRNA. Variants G1896A and G1899A, on the other hand, impact the base pairing of the stem structure within the epsilon region of the pgRNA^16,19–21^, affecting the secondary RNA structure and consequently the encapsidation of the pgRNA, a crucial step prior to the generation of the rcDNA.

### Viral variants associated with patient HBeAg status

**Figure 2:**
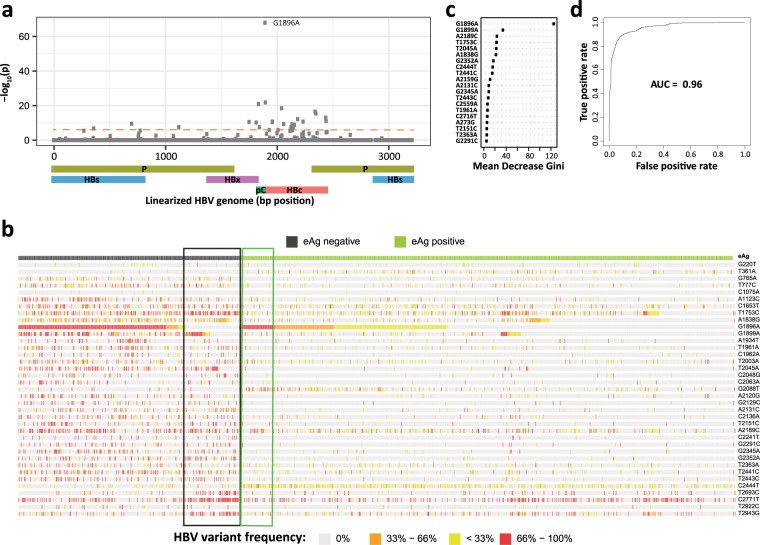
HBV variants associated with patient’s HBeAg status. (**a**) Manhattan plot showing an association (Likelihood Ratio Test *p* value, see methods) between HBV variants at a given genomic position and patient’s HBeAg status. Bonferroni correction results in a significance threshold of approximately 1.6 × 10^-6^ indicated by the red dashed line. See Supplementary Table 3 for a detailed list of variants associated with HBeAg status. Schematic below x axis represents HBV genome structure. P – polymerase, S – surface protein, C – core protein, pC – pre-core region, X – HBx protein. (**b**) Heatmap of HBV variants associated with HBeAg status and detected across 1102 patients of the discovery cohort. The 37 variants selected for classification modeling are displayed. The top row indicates patient’s HBeAg status. Columns (patients, N = 1102) are ordered by HBeAg status and by G1896A frequency. Rows (HBV variants) are ordered given variant’s genome position. Black box highlights HBeAg negative patients without detectable viral G1896A variant. The green box highlights HBeAg positive patients with a high frequency of viral G1896A variant. (**c**) Mean decrease in Gini index, reflecting a relative importance of a variant within a HBeAg status classifier model, is shown for top 20 variants. See Supplementary Table 3 for a detailed list of variants used in the model and their mean decrease in Gini index. (**d**) ROC and AUC from random forest model classifying patient’s HBeAg status within the discovery patient cohort (N = 1102).

The next clinical phenotype we found to be strongly associated with certain HBV variants was patient HBeAg status. The variant with the strongest association was G1896A (LRT; *p* = 5.43 × 10^−69^, Fig. 2a**)**. Aside from its previously mentioned role in the epsilon region of the pgRNA, G1896A introduces a stop codon in the precore region abolishing HBeAg production^10–15^. The confirmation of this well-known mutation validates our approach. More importantly, we found that G1896A is not a necessary variant determining patient’s HBeAg status. Despite deep sequencing coverage at the G1896A locus (median_coverage_ = 8949), a substantial portion of 24% (22% excluding HBV genotype A, see Discussion) of HBeAg-negative patients had undetectable frequency of G1896A variant (Fig. 2b).

Our unbiased approach led us to the discovery of additional variants highly associated with patient’s HBeAg status: G1899A, A1838G, T2045A, G2345A, G2352A, T2441C, A2189C, T2443C, C1962A, G2237C, and others (LRT; *p* < 9.55 × 10^−13^; Fig. 2b,c; see Supplementary Table 3 and Fig. 5). To further evaluate the relevance of G1896A in conjunction with other variants in determining patient HBeAg status, we built several classification models to assess the collective predictive power and rank the significance of each variant. In fact, a classification model based solely on G1896A frequency attains only a relatively modest performance predicting HBeAg status with a low true negative rate (Specificity = 0.60, AUC = 0.81). This observation is largely explained by the aforementioned fact that 24% of HBeAg-negative patients lacked G1896A whereas 7.3% of HBeAg-positive patients had G1896A at higher than 0.66 in frequency (Fig. 2b).

Next, we assessed the predictive power of additional variants by building a random-forest classifier using a total of 37 significant variants, including G1896A (see methods for viral variant selection). We found that inclusion of additional variants yields a significantly better classification performance (specificity = 0.89, accuracy = 0.92, precision = 0.95, sensitivity = 0.93, AUC = 0.96, Fig. 2c,d). To address potential overfitting issues, this classification model was applied to the independent validation patient cohort and yielded a comparable performance (specificity = 0.97, accuracy = 0.93, precision = 0.95, sensitivity = 0.87, AUC = 0.92). Although G1896A frequently disrupts the production of HBeAg via the introduction of a premature stop codon, other mutations are likely to achieve a similar clinical effect via different molecular means.

**Figure 3:**
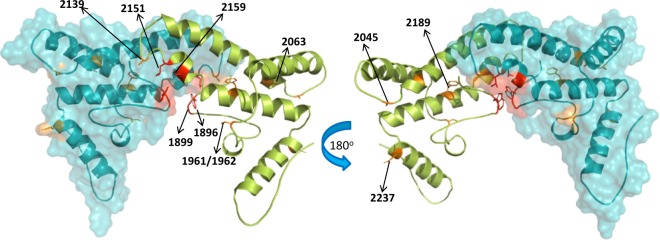
HBeAg dimer structure. Two views of the HBeAg dimer structure (PDB: 3V6Z) with one monomer in teal surface representation and the other in green cartoon representation. Shown is a subset of HBV variants labeled by HBV genome position. The four HBeAg residues harboring 1896, 1899, 2151, and 2159 variants are at the dimer interface and are colored in red; six HBeAg residues harboring 1961/1962, 2045, 2063, 2139, 2189, and 2237 variants are within intra-dimer interaction region and are colored in orange.

In order to further understand the potential functional impacts of these variants on HBeAg status, we overlaid these variants onto the HBeAg 3D crystal structure^22^ (Fig. 3). The location of these variants suggests potential disruption of the HBeAg dimer. For example, variant G1899A induces Gly to Asp change (Gly/Asp29) in a loop at the dimer interface interacting with the same residue from the other dimer at a distance of ~6 Å. Mutation to a bigger and acidic aspartate likely induces charge-charge repulsion resulting in destabilization of the observed dimer form. Variant G2237C corresponds to Glu/Gln142 located at the end of an α-helix, forming a strong salt-bridge interaction with Arg141 and stabilizing the domain structure in this region. This mutation likely destabilizes this strong interaction leading to structural defects. While the association between G1896A and patient HBeAg status is highly significant, the presence of G1896A alone cannot completely explain patient HBeAg status. Our data therefore suggest that, in the absence of G1896A, combinations of other variants are likely interfering with HBeAg production or protein structure resulting in HBeAg negative phenotype.

### Association of other clinical characteristics with viral variants

We also investigated possible associations between HBV variants and HBsAg and ALT levels, fibrosis, and a loss of HBsAg. We either did not find a strong association for these clinical factors in the discovery patient set or the initial association was not validated in the independent cohort.

## Discussion

This analysis, which systematically inspected the whole HBV genome for variants in a large patient population, identified novel HBV genetic factors of viral load, which is one of the strongest independent risk predictors for disease progression, cirrhosis, and increased mortality from HCC and chronic liver disease^23–30^. Interventional studies demonstrated that reduction of the viral load strongly correlated with an improvement in liver histology^23,31–33^. Our data show that increased frequency of viral variants C1817T and A1838G were associated with significantly lower serum levels of HBV DNA at baseline.

Due to the extremely compact and functionally overlapping architecture of the HBV genome, it is challenging to determine the precise mechanism of action for these variants. Some clues can be taken from an elegant study by Lewellyn and Loeb^34^ who identified cis-acting sequences contributing to the template switching during the plus-strand DNA synthesis of HBV. In particular, h3E and hM regions are in close proximity with each other and facilitate primer translocation and circularization of relaxed-circular (rc) DNA, an essential step of viral replication. The A1838G variant is located within this h3E region where nucleotide substitutions in the *in vitro* model resulted in a significant decrease in the production of rcDNA^16^. These experimental results are in keeping with our observation that patients carrying this variant in the h3E region display significantly reduced viral load when compared to wildtype.

The C1817T variant lies in the second codon of precore inducing a stop codon. Coincidentally, this variant also resides directly upstream of the pgRNA which is a precursor to the HBV rcDNA. Given its proximity to the transcription start site, we hypothesize that C1817T affects the transcriptional regulation of pgRNA leading to the reduction of the viral load. Our association findings and this experimental evidence suggest these two variants likely utilize two different molecular mechanisms through which they negatively impact viral load - modification of h3E region involved in plus-strand DNA synthesis and a regulatory effect on pgRNA transcription.

Although patients with high frequency of C1817T and A1838G variants experienced earlier viral suppression while on treatment, given the effectiveness of the latest reverse transcriptase inhibitors, the difference in viral load reduction diminishes after 48 weeks. We believe that monitoring of these specific variants in clinical trials investigating the efficacy of drugs with other mechanisms of action (such as Toll-Like Receptor Agonists, immune checkpoint inhibitors, therapeutic vaccines, capsid/core inhibitors, etc.) may be more relevant to clinical practice.

Next, our data is consistent with the hypothesis that change in patient’s HBeAg status is primarily a mutation-driven process. Whether the HBeAg mutations are driven by the error-prone HBV polymerase or by the host innate immune system through Apolipoprotein B mRNA Editing Catalytic Polypeptide-like gene family (APOBEC), for example, the integrity of the HBeAg coding sequence seems to be the determining factor. Although G1896A had the most significant association with HBeAg status, we identified other variants that were important for understanding a patient’s HBeAg status. While G1896A disrupts the production of HBeAg via a premature stop codon in the precore region, other variants likely inhibit dimerization or protein folding of HBeAg. This observation further highlights the intriguing diversity of the molecular mechanisms through which viral variants affect a specific clinical phenotype. It is worth mentioning that G1896A variants in HBV genotype A and some genotype C strains are rare due to genotype-specific constraints in the base-pairing of the pgRNA secondary structure^20,35^ and other mutations become solely responsible for the disruption of HBeAg production. Previous studies described additional variants in the core promoter including C1653T, T1753C, A1762T, and G1764A and their correlation with the downregulation of precore mRNA^36,37^. The reported clinical impact of these variants entailed associations with HBeAg status; however, these connections have not always been consistent across all studies^5,38–44^. While our study did not identify any significant association with A1762T and G1764A, we found T1753C to be strongly associated with HBeAg status in our dataset (Supplementary Table 2). C1653T exhibited only marginal signal with viral load.

HBsAg is a hallmark serological marker of HBV infection and its reduction to undetectable levels along with patient’s development of anti-HBsAg antibodies (HBsAg seroconversion) is clinically considered a functional cure. Uncovering viral variants associated with HBsAg levels would be of tremendous interest to both scientists and clinicians, however, we did not observe any consistent associations. This finding is in line with clinical observations that it is very challenging to lower serum levels of HBsAg despite successful nucleoside inhibition of viral replication. One hypothesis would be that integrated HBV DNA significantly contributes to the serum levels of HBsAg, as has been shown in chimpanzees^45^, which would confound any possible association with viral variation.

This work adds to the current knowledge of associations between viral genetic factors and patient clinical characteristics. Future employment of long-read technology capable of spanning the entire ~3.2Kb HBV genome could help us further resolve mutation linkage and give us insights into the joint effect of multiple variants. As this study examined associations between viral variation and clinical parameters, future investigations combining human genetic variations might provide additional perspective on viral-human genetic interaction and co-evolutions^46,47^. In summary, our data not only identifies novel variants associated with major clinical factors but will also serve as a resource to the research and medical community where novel treatment strategies (e.g. development of HBV surface/capsid inhibitors and DNA editing approaches) depend on the understanding of viral sequence variation across global patient cohorts.

## Materials and Methods

### Ethics statement and patient samples

Baseline serum samples analyzed in this study came from 1102 patients enrolled in two global phase 3 studies of tenofovir alafenamide (TAF) versus tenofovir disoproxil fumarate (TDF) for the treatment of HBeAg-negative and -positive chronic hepatitis B (GS-US-320-0108 and GS-US-320-0110) and 365 patients enrolled in GS-US-174-0149 clinical trial, evaluating TDF and peg-interferon alpha-2a combination therapy in treatment-naive patients chronically infected with HBV. Patient samples that were part of this analysis were collected from 21 countries (Australia, Canada, France, Germany, Greece, Hong Kong, India, Italy, Japan, Netherlands, New Zealand, Poland, Romania, Russia, Singapore, South Korea, Spain, Taiwan, Turkey, United Kingdom, and United States). All patients signed an informed consent form prior to screening and in accordance with local regulatory and ethics committee requirements. Experimental protocol in these trials was approved by Gilead Sciences and all local regulatory agencies (see ClinicalTrials.gov: NCT01277601, NCT01940341, and NCT01940471 for trials GS-US-174-0149, GS-US-320-0108, and GS-US-320-0110, respectively).

### HBV genome sequencing and mapping

DNA isolation was performed using Qiagen MinElute kit for serum samples with viral load <100,000 IU/mL and Roche MagNA Pure robot 32 for samples with viral loads > 100,000 IU/ml. HBV whole genome was amplified following Gunther *et al*.^48^. Whole genome HBV amplicons were sequenced on Illumina MiSeq with 150 bp paired-end reads. Low quality bases (Q < 20) at 5′ and 3′ of each read were trimmed with Trimmomatic (v0.35) and reads shorter than 50 bp were removed. Subsequently, paired reads were merged based on overlapping regions and sequencing error correction was performed using PEAR (v0.9.6). Unmerged reads were discarded. Read mapping was performed with BWA (0.7.9a) and the reference genome for each sample was chosen from HBVdb.ibcp.fr^49^ given patient’s genotype, which was determined via laboratory genotyping assay. Accession numbers: genotype A – EU054331, genotype B – AB219428, genotype C - GQ924620, genotype D – FJ904433. It is important to note that these genotype specific reference sequences were only used to facilitate sequence alignment and were not used for variant calling. Therefore the selection of specific HBV reference sequences does not impact what constitutes a reference or variant allele (see Variant calling and calculating wildtype frequency section for more details).

### Variant calling and calculating wildtype frequency

Because collapsing viral genomes within each patient into a single viral consensus sequence will remove potentially valuable genetic information, we calculated wildtype frequency for each viral genome position and for each patient. Since several HBV genotypes can be genetically more than ~10% divergent, determining wildtype allele across all genotypes is not plausible and, furthermore, may also remove information for loci with more than two alleles. We therefore calculated wildtype frequency by calling variants from a genotype consensus generated from across our HBeAg positive patient population. This approach also partially controls for genotype specific effects during association testing. We chose HBeAg positive patients for genotype consensus reconstruction because this disease stage represents an earlier phase of infection. After read mapping, we generated primary consensus sequence (based on highest allele frequency) with custom scripts for each HBeAg positive patient. Primary consensus sequences were pooled given the HBV genotype determined from clinical assay and for each genotype we generated a secondary consensus. Because the depth of coverage of our sequencing experiments was very high (often exceeding 9000x coverage) across large patient cohort (977 HBeAg positive patients), the primary and secondary consensus call could always be made implementing the majority rule since no two variants within a patient or across patients had exactly the same frequency. Using an appropriate genotype specific secondary consensus, we called variants for each patient (both HBeAg positive and negative) using samtools mpileup^50^ (version 1.3). The parameters for base pair and mapping quality were -Q 20 -q 20 -d 50000 and variants were subsequently filtered with varscan^51^ (version 2.3.9) package given parameters for minimum number of reads, minimum frequency, and minimum p value (–min-reads2 5–min-var-freq. 0.02–p-value 0.1–min-coverage 100–min-avg-qual 25). We further removed variants with a strand bias greater than 5. Assessing sequencing benchmarks from control plasmids and experimenting with minimum variant frequency cutoffs, we applied a minimum variant frequency cutoff of 2% to eliminate any background noise introduced via PCR and sequencing errors.

### Calculating association between viral variants and clinical traits

Associations between viral variants (viral wildtype frequency) and patient’s clinical variables were analyzed using Generalized Linear Models (GLM) while adjusting for appropriate co-factors. Specifically, for each response variable tested, we compared m_0_ (model without HBV variant frequency co-factor) with m_1_ (model with HBV variant frequency co-factor). Likelihood Ratio Test between m_0_ and m_1_ was performed to assess whether addition of HBV variant frequency significantly improved model fit. Models of clinical traits such as ALT, HBsAg levels, and HBV DNA levels were adjusted for HBV genotype, gender, age, race, and HBeAg status. Models of HBsAg and HBV DNA treatment response were adjusted for HBV genotype, gender, race, age, HBeAg status, treatment arm, and treatment experience. Model of HBeAg status was adjusted for HBV genotype, gender, race, and age. Models of HBeAg and HBsAg loss were adjusted for HBV genotype, gender, race, age, baseline levels of HBV DNA and HBsAg, treatment arm, and treatment experience. Multiple testing correction via Bonferroni method led to a significance threshold of approximately 1.6 × 10^−6^. All modeling was performed independently on discovery and validation sets of patients. Associations significant in both cohorts are reported here and became candidates for further exploration.

### Predictive modeling of HBeAg status

To investigate how well HBV variants can predict patient’s HBeAg status, we built a random forest classification model. Because the number of features (N = 2281) greatly exceeded the number of patients (N = 1102) in the discovery cohort, we performed a feature selection assisted with Elastic Net method^52^. Specifically, we combined top 10 variants individually found to be associated with HBeAg status with variants selected by Elastic Net, which explained at least ~50% of HBeAg status variation. The reason for this strategy was two-fold. First, we wanted to select top variants by the fraction of HBeAg status variation they collectively explain, rather than by choosing specific association p value cutoff. Second, we decided to supplement the selection of top variants supplied by Elastic Net due to a known behavior, where one of two highly correlated variants gets dropped by Elastic Net. This approach yielded 37 variants. Using these 37 variants we then built a random forest model (number of trees to grow = 2000) for the classification of patient’s HBeAg status. Validation of the random forest model was performed on an independent set of patient data (N = 365).

### Site directed mutagenesis and transient transfection system assay

Site directed mutants (SDM) were created for the C1817T and A1838G mutations, both individually and in combination. Primers were designed for the SDM mutagenesis process in order to introduce the substitutions into a plasmid vector containing the HBV genotype A 1.1x genome length laboratory strain pHY92 under the control of a CMV promoter. The entire HBV genome of the construct was sequenced to confirm that no additional mutations had been introduced during the SDM process. Intracellular and extracellular HBV DNA were assessed 7 days after transient transfection of the SDM and pHY92 constructs into HepG2 cells (ATCC, VA) using FuGENE 6 (Promega, WI). HBV DNA in the supernatant was quantified using the branched DNA technology QuantiGene® 2.0 (Thermo Fischer Scientific, MA) per manufacturer’s instructions. HBV DNA in the cells was quantified using quantitative PCR specific to the HBV X gene. For SDM constructs, fold changes in HBV DNA production with respect to the corresponding reference sample pHY92 were reported. The transfection experiments were performed only in the pHY92 genotype A background context and may not fully extrapolate to other HBV genotypes. However, given the comparatively lower replication rate of genotype A strains^53^, this experiment may reflect a conservative estimate of mutation effect on HBV DNA production.

### HBeAg 3D structure

The structure of HBeAg dimer was downloaded from the Protein Data Bank (www.rcsb.org). The structure was prepared using the ProteinPrep utility in Maestro (www.schrodinger.com) and visualizations and analysis were generated using Pymol (www.schrodinger.com). The residue numbering in the discussion is offset by +19 from the residue numbering in the X-ray.

## Supplementary information

SUPPLEMENTARY INFORMATION

## Data Availability

All sequencing files with associated metadata included in this study were deposited at EGA European Genome-Phenome Archive (ega-archive.org) under accession code EGAS00001003689.
