# Supplementary material for: Large-scale viral genome analysis identifies novel clinical associations between hepatitis B virus and chronically infected patients
Source: Sci Rep. 2019 Jul 19;9:10529. doi: 10.1038/s41598-019-46609-7 (PMC6642195; doi:10.1038/s41598-019-46609-7)
Supplement: Supplementary file 1 — SUPPLEMENTARY INFORMATION [file 41598_2019_46609_MOESM1_ESM.pdf]

Supplementary Information

Large-scale viral genome analysis identifies novel clinical associations between hepatitis B virus and chronically infected patients

Ondrej Podlaha[1*], Edward Gane[2], Maurizia Brunetto[3], Scott Fung[4], Wan-Long Chuang[5], Calvin Pan[6], Zhaoshi Jiang[1], Yang Liu[1], Neeru Bhardwaj [1], Jit Mukherjee [1], John Flaherty[1], Anuj Gaggar[1], Mani Subramanian[1], Namiki Izumi[7], Shalimar[8], Young Suk Lim[9], Patrick Marcellin[10], Maria Buti[11], Henry LY Chan[12], Kosh Agarwal[13].

[1] Gilead Sciences Inc., 333 Lakeside Drive, Foster City, CA, 94404, USA
[2] Auckland Clinical Studies, Auckland, New Zealand
[3] Internal Medicine, Department of Clinical and Experimental Medicine, University of Pisa, Pisa, Italy and Liver Unit, University Hospital of Pisa Hepatology Unit, University Hospital of Pisa, Pisa, Italy
[4] Toronto General Hospital, Toronto, ON, Canada
[5] Kaohsiung Medical University Hospital, Kaohsiung Medical University, Kaohsiung, Taiwan
[6] Division of Gastroenterology and Hepatology, Department of Medicine, NYU Langone Medical Center, NYU School of Medicine, New York, NY, USA
[7] Department of Gastroenterology and Hepatology, Musashino Red Cross Hospital, Tokyo, Japan
[8] All India Institute of Medical Sciences; Department of Gastroenterology New Delhi, India
[9] Department of Gastroenterology, Asan Medical Center, Seoul, South Korea
[10] Service d'Hépatologie, Hôpital Beaujon, Clichy, France
[11] Liver Unit, Department of Medicine, Hospital General Universitari Vall d'Hebron and Ciberehd del Instituto Carlos III, Barcelona, Spain
[12] The Chinese University of Hong Kong, Hong Kong
[13] Kings College Hospital, London, UK
[*] Corresponding author

Prevalence of C1817T and A1838G viral variants and viral load across HBV genotypes

The prevalence and co-occurance of C1817T and A1838G viral variants varies across HBV genotypes (**Supplementary Table 1**). This observation is however confounded by an uneven representation of the major HBV genotypes in our patient cohorts (enrolled in GS-US-174-0149, GS-US-320-0108, and GS-US-320-0110 clinical trials), where genotypes C (N=716), D (N=356), and B (N=285) were most predominant, while genotype A (N=98) was under-represented. Although the frequency of C1817T was 10.8% across all patients, the frequency of C1817T in genotype A was only 5.6% and conspicuously missing in genotype D. Additionally, C1817T rarely occurs alone (0.4% across all genotypes) making it challenging to investigate its sole association with baseline serum levels of HBV DNA. A1838G variant, on the other hand, had more even distribution of frequencies across major HBV genotypes with an overall frequency of 17.1%. It is worth noting that both variants C1817T and A1838G are ~4.5 times more frequent in HBeAg-negative than HBeAg-positive patients. Supplementary Figure 2 displays baseline viral loads across major HBV genotypes, HBeAg status, and combinations of viral variants C1817T and A1838G. As is seen from this figure, wherever there is a large enough sample size (N>10), the differences in baseline viral load between patients carrying C1817T and A1838G viral variants and wildtype is always statistically significant (Wilcoxon rank-sum test, $p < 0.05$). Due to the relatively low prevalence of C1817T, a larger patient population is needed to gain a robust understanding of this variant specifically in genotype A, and genotype B HBeAg positive patients.

Relationship between C1817T, A1838G, G1896A viral variants and baseline viral load

Given the significant association between C1817T and A1838G and baseline serum HBV DNA, we further investigated these variants in the background of G1896A, which is a well-known precore stop codon mutation correlated with negative HBeAg status as well as lower viral load. Categorizing patients based on G1896A variant frequency (categories: 0%, <0%-33%, 33%-66%, 66%-100%) shows that i) C1817T, A1838G variants are present in all G1896A background categories, and ii) patients with C1817T or A1838G viral variants have significantly lower viral load across all G1896A background categories (**Supplementary Figure 2**).

Supplementary Table 1. **Prevalence of high-frequency C1817T and A1838G mutations across the patient population by HBV genotype.** Intra-patient high-frequency mutations were based on naturally occurring frequency cut-off of 10% (see main text and Supplementary Figure 1).

	Frequency (%)					
**HBV genotype**	**C1817T alone**	**A1838G alone**	**C1817T and A1838G**	**C1817T or A1838G**	**at least 1817**	**at least 1838**
A (N = 98)	0	0.155	0.056	0.211	0.056	0.211
B (N = 285)	0.005	0.015	0.128	0.148	0.133	0.143
C (N = 716)	0.005	0.009	0.15	0.165	0.155	0.159
D (N = 356)	0	0.2	0	0.2	0	0.2
E (N = 7)	0	0	0.429	0.429	0.429	0.429
F (N = 5)	0	0.2	0	0.2	0	0.2
All genotypes (N = 1467)	0.004	0.067	0.104	0.175	0.108	0.171
All genotypes HBeAg+ (N = 977)	0	0.031	0.052	0.083	0.052	0.083
All genotypes HBeAg- (N = 490)	0.012	0.148	0.227	0.387	0.239	0.375

Supplementary Table 2. The median difference in viral load (serum HBV DNA levels) between wildtype and HBV variants (C1817T and A1838G) associated with patient viral load given a range of variant frequency cutoffs. These tests show that significant differences in viral load are not sensitive to the range of frequency cutoff values determining high and low variant frequency patient groups.

**Variant**	**Frequency cutoff**	**Median difference in viral load from wildtype (log_{10}IU/mL)**	**Wilcoxon signed-rank test (*P*)**
A1838G alone	0.03	2.75	2.40E-27
A1838G alone	0.04	2.67	2.62E-24
A1838G alone	0.05	2.73	3.25E-24
A1838G alone	0.06	2.73	3.97E-24
A1838G alone	0.07	2.59	1.30E-24
A1838G alone	0.08	2.60	5.06E-25
A1838G alone	0.09	2.60	5.06E-25
A1838G alone	0.1	2.60	1.64E-25
A1838G alone	0.11	2.67	4.70E-26
A1838G alone	0.12	2.67	4.70E-26
A1838G alone	0.13	2.67	4.70E-26
A1838G alone	0.14	2.67	5.88E-27
A1838G alone	0.15	2.46	2.70E-27
A1838G alone	0.16	2.46	8.05E-28
A1838G alone	0.17	2.57	7.57E-29
A1838G alone	0.18	2.59	2.93E-30
A1838G alone	0.19	2.66	2.52E-31
A1838G alone	0.2	2.74	3.22E-34
A1838G alone	0.21	2.79	3.06E-36
A1838G alone	0.22	2.81	3.41E-37
A1838G alone	0.23	2.81	1.02E-37
A1838G alone	0.24	2.81	2.01E-40
A1838G alone	0.25	2.81	8.80E-43
C1817T & A1838G	0.03	2.75	3.58E-57
C1817T & A1838G	0.04	2.72	5.20E-56
C1817T & A1838G	0.05	2.73	8.38E-56
C1817T & A1838G	0.06	2.73	1.21E-55
C1817T & A1838G	0.07	2.77	1.26E-54
C1817T & A1838G	0.08	2.80	2.95E-54
C1817T & A1838G	0.09	2.80	2.95E-54
C1817T & A1838G	0.1	2.80	7.00E-54
C1817T & A1838G	0.11	2.77	2.21E-53
C1817T & A1838G	0.12	2.75	7.14E-53
C1817T & A1838G	0.13	2.75	7.14E-53
C1817T & A1838G	0.14	2.75	4.65E-52
C1817T & A1838G	0.15	2.84	4.98E-52
C1817T & A1838G	0.16	2.84	2.88E-51
C1817T & A1838G	0.17	2.82	2.75E-50
C1817T & A1838G	0.18	2.80	6.06E-49
C1817T & A1838G	0.19	2.75	6.63E-48
C1817T & A1838G	0.2	2.70	4.38E-45
C1817T & A1838G	0.21	2.68	4.51E-43
C1817T & A1838G	0.22	2.67	3.53E-42
C1817T & A1838G	0.23	2.67	1.19E-41
C1817T & A1838G	0.24	2.61	6.59E-39
C1817T & A1838G	0.25	2.51	1.82E-36

Supplementary Table 3. List of 37 mutations selected as random forest model input for classifying patient's HBeAg status. See methods for information on feature selection. LRT p value represents the Likelihood Ratio Test between a set of models assessing HBV variant association with patient's HBeAg status. Mean decrease in gini reflects relative importance of a variant within an HBeAg status classifier model.

HBV variants	LRT p value	Mean decrease gini
G1896A	3.85E-73	128.36
G1899A	5.08E-27	33.30
A1838G	4.17E-26	23.32
T2045A	1.27E-23	22.88
G2345A	1.21E-21	9.30
G2352A	1.31E-20	18.19
T2441C	3.38E-18	15.77
A2189C	2.56E-17	23.73
T2443C	3.25E-16	8.55
C1962A	6.09E-16	2.74
G2237C	7.11E-16	4.59
T1753C	9.63E-16	22.37
T1961A	1.17E-15	6.83
C2063A	1.71E-14	4.00
A2159G	4.34E-14	12.20
T2151C	5.61E-14	6.06
A1123C	1.14E-12	5.38
G2129C	1.14E-12	4.15
A2131C	7.52E-12	9.67
A1934T	4.55E-11	4.30
C2136A	6.29E-11	5.05
T777C	1.08E-10	5.29
A273G	1.33E-10	6.55
A2120G	1.49E-10	3.50
G2452A	5.85E-10	3.64
C2444T	1.32E-09	17.19
T2363A	2.82E-09	6.16
G2291C	5.51E-09	5.26
C2048G	6.00E-08	2.68
C919A	8.47E-08	1.52
C2241T	2.53E-07	2.84
T2943G	3.24E-06	5.40
C2559A	3.36E-05	7.26
T2693C	1.24E-04	4.28
G2088T	2.08E-04	3.55
C539A	1.02E-03	5.21
C2716T	2.33E-03	7.22

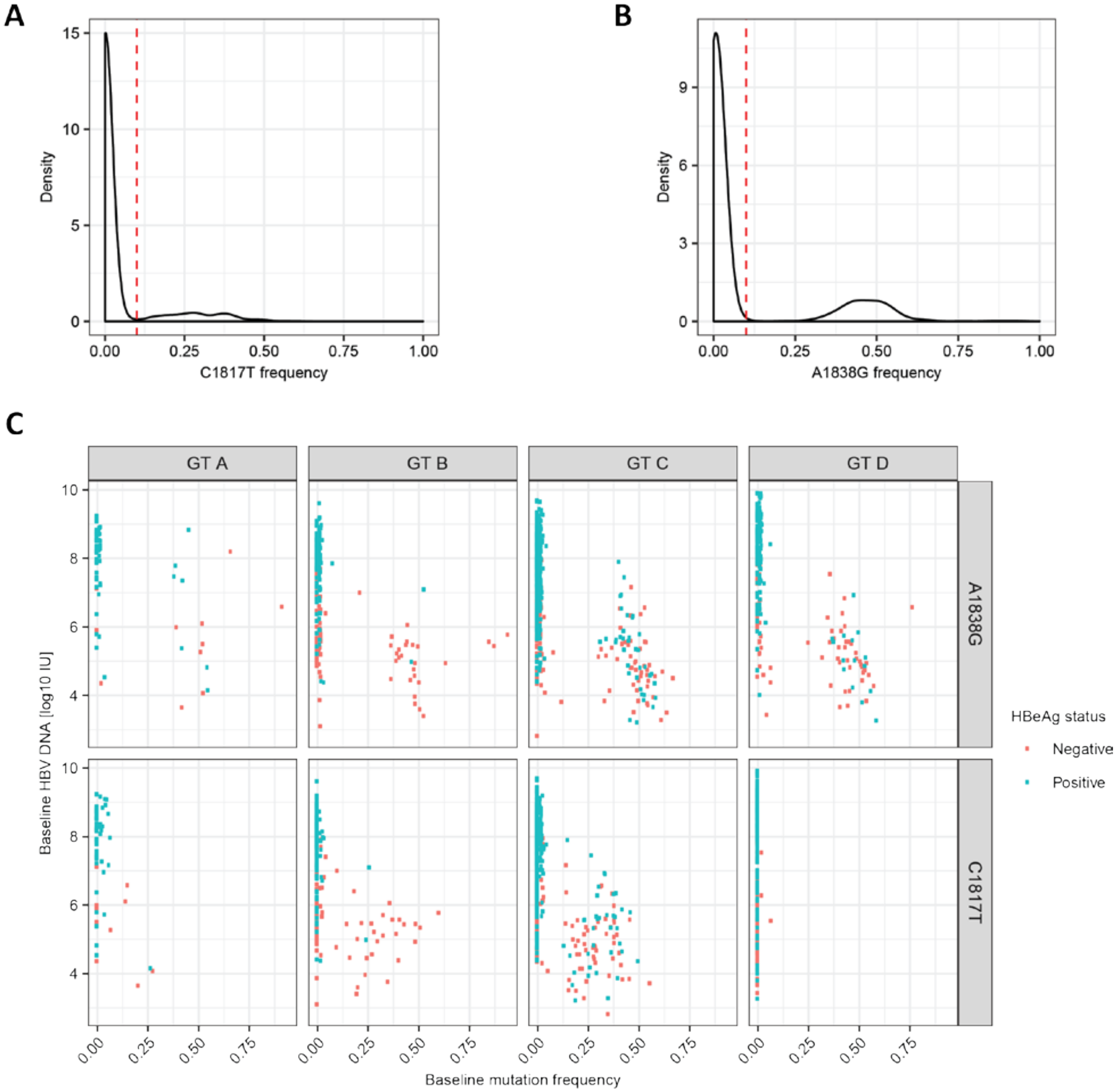

Supplementary Figure 1. Density plots of HBV variants associated with viral load. The frequency distribution of C1817T (**A**) and A1838G (**B**) displayed a bimodal distribution allowing patients to be broadly divided into high and low frequency groups given a 10% frequency cutoff (vertical dashed red line). Patients in the high frequency group displayed significantly lower levels of viral load. To assess the sensitivity of our observations to a given cutoff, range of cutoffs between 5%-20% were also tested (see **Supplementary Table 2**). (**C**) A detailed breakdown of C1817T and A1838G frequencies across four major HBV genotypes (A, B, C, D) and HBeAg status.

A

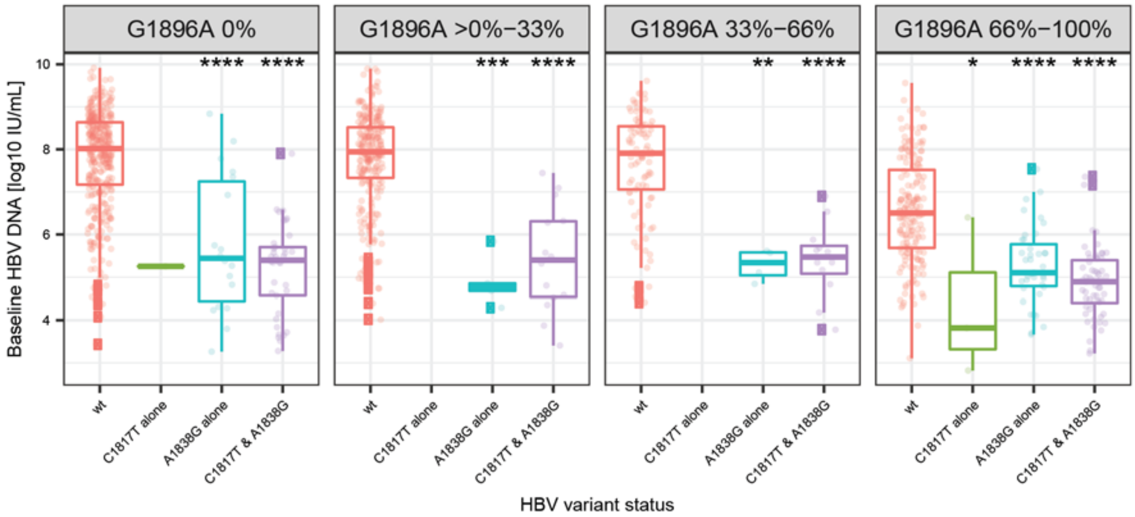

B

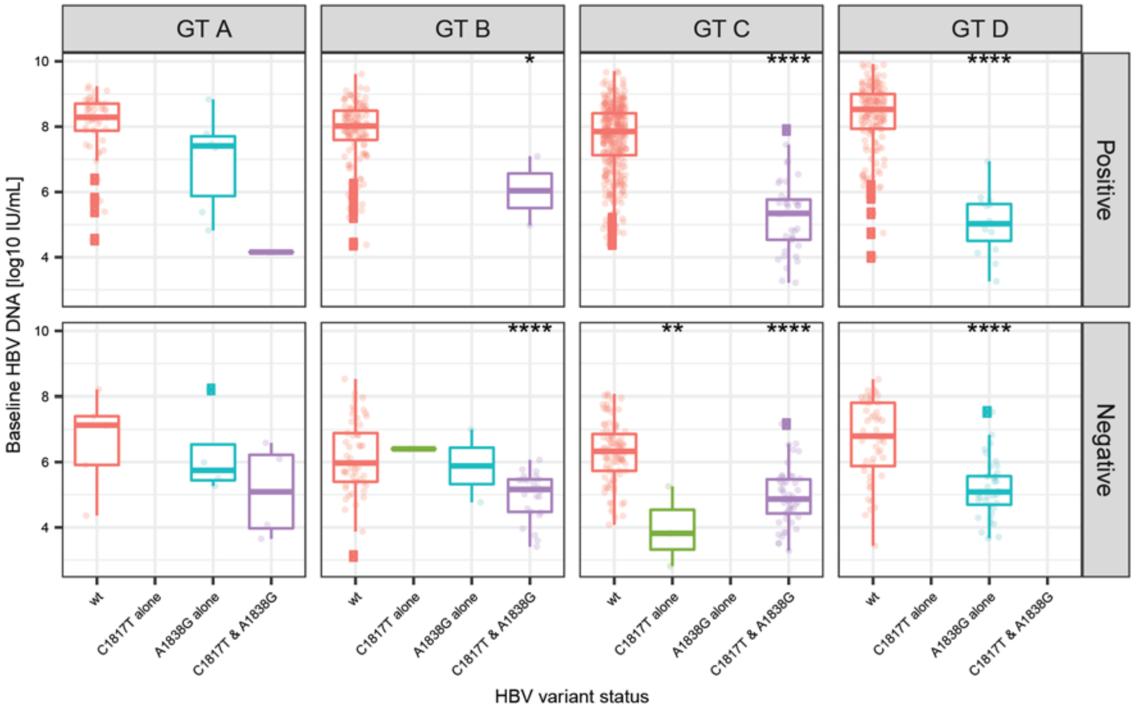

Supplementary Figure 2. C1817T and A1838G variant association with HBV DNA levels in the background of G1896A and HBeAg positive and negative patients. Association of variants, individual or in combination, with patient's viral load (serum levels of HBV DNA). (**A**) G1896A is a well-known precore stop codon mutation correlated with negative HBeAg status as well as a lower viral load. To understand how is C1817T and A1838G associated with baseline viral load given G1896A background, we categorizing patients based on G1896A variant frequency (categories: 0%, <0%-33%, 33%-66%, 66%-100%). C1817T, A1838G variants are present in all G1896A background categories, and patients with C1817T or A1838G viral variants have significantly lower viral load across all G1896A background categories. (**B**) We further plotted baseline viral load given C1817T and A1838G across four major HBV genotypes

and patient's HBeAg status. Wherever clinical subsets have a large enough sample size (N>10), the differences in baseline viral load between patients carrying C1817T and A1838G viral variants and wildtype is always statistically significant. Due to the relatively low prevalence of C1817T, a larger patient population is needed to gain a robust understanding of this variant specifically in genotype A, and genotype B HBeAg positive patients.
Wilcoxon rank sum test: *$p < 0.05$; **$p < 0.01$; ***$p < 0.001$, ****$p < 0.0001$.

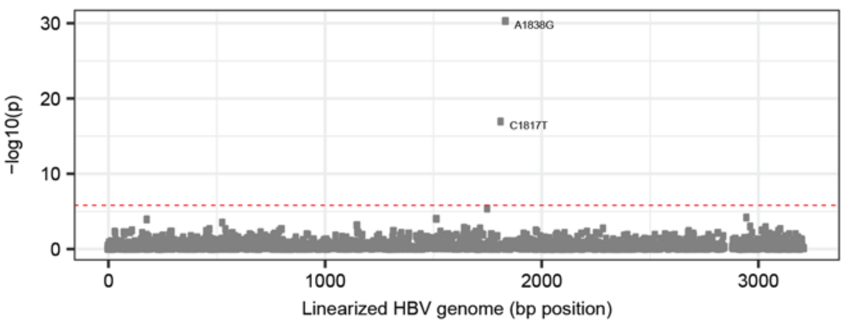

Supplementary Figure 3. HBV variants associated with patient’s HBeAg status in the validation cohort. Manhattan plot showing an association between HBV variants at a given genomic position and patient’s HBeAg status in the validation cohort (N = 365). Bonferroni correction results in a significance threshold of approximately 1.6 x 10^{-6} indicated by the red dashed line. The x axis represents the HBV genome position starting with EcoR1 site.

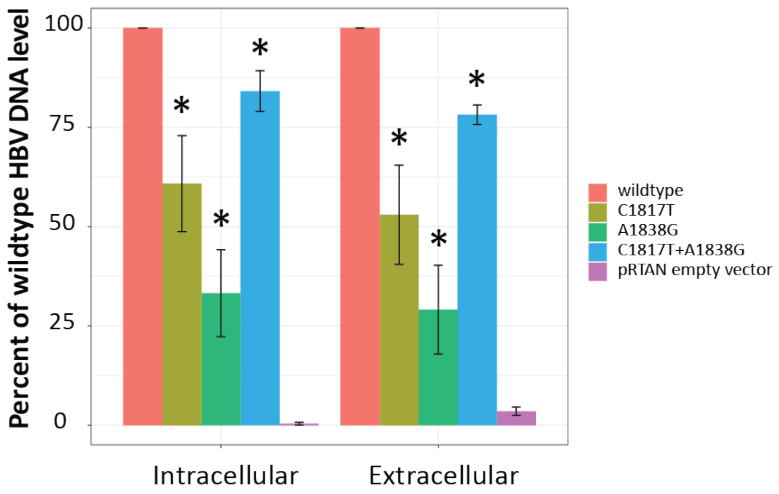

Supplementary Figure 4. Effect of C1817T and A1838G on HBV DNA levels in a transient transfection system. Experimental validation of C1817T and A1838G variants in a transient transfection system - pHY92 strain generated from genotype A hepatitis B virus – measuring intracellular and extracellular HBV DNA production. Individually, C1817T and A1838G reduced extracellular HBV DNA by 47.0% and 70.1% of wildtype expression, respectively. Combination of mutation C1817T and A1838G brought down extracellular HBV DNA by 21.8% of wildtype. Reduction of extracellular HBV DNA in this transient transfection system is concordant with our observations in patient cohorts. Further investigation is however needed to fully understand the effect of mutation combination. One-tailed Students t-test * $p<0.05$; ** $p<0.01$; *** $p<0.001$.

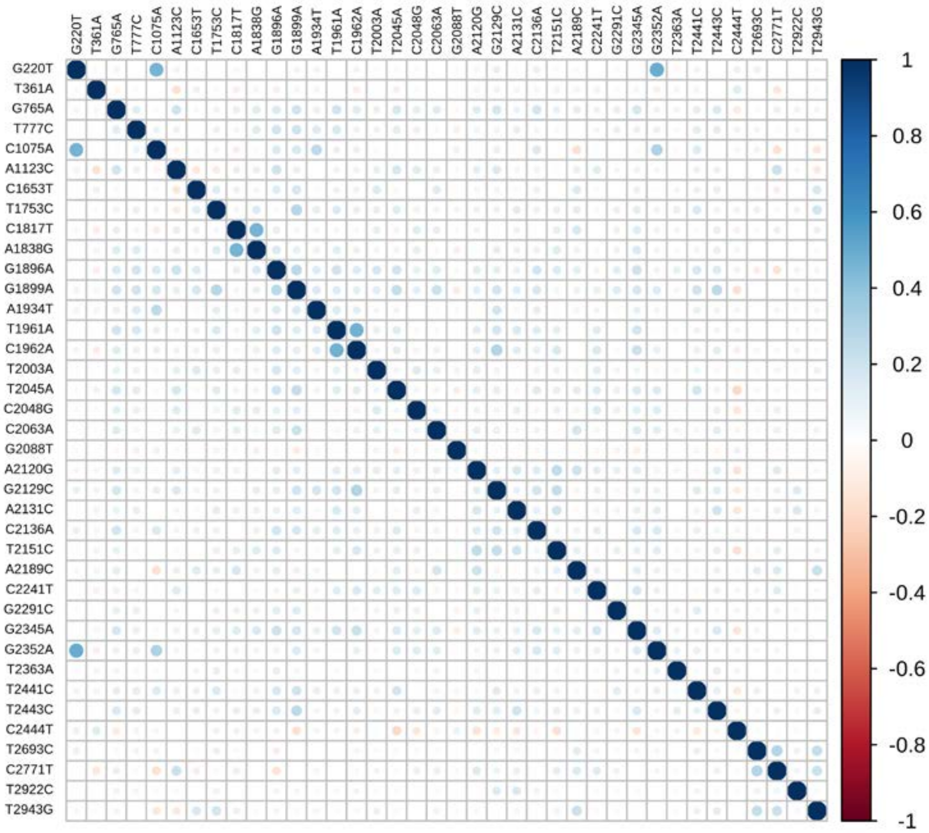

Supplementary Figure 5. Matrix of Spearman rank correlation coefficients between all viral variants used to build an HBeAg classification model and C1817T and A1838G. Because the variants used in the Random Forest classification model were identified by a feature selection process (described in the methods section) to specifically classify patient's HBeAg status, these variants are expected to have minimal informational redundancy, and do not frequently co-occur together (C1817T was not selected for the classification model). The vast majority of these variants show minimal correlation with each other and A1838G. This observation doesn't mean; however, that these variants are mutually exclusive, but rather that they independently associate with HBeAg status.
